# Pyrethroids resistance intensity and resistance mechanisms in *Anopheles gambiae* from malaria vector surveillance sites in Nigeria

**DOI:** 10.1371/journal.pone.0205230

**Published:** 2018-12-05

**Authors:** Taiwo Samson Awolola, Adedapo Adeogun, Abiodun K. Olakiigbe, Tolulope Oyeniyi, Yetunde Adeola Olukosi, Hilary Okoh, Tolulope Arowolo, Joel Akila, Adedayo Oduola, Chioma N. Amajoh

**Affiliations:** 1 Nigerian Institute of Medical Research, 6 Edmund Crescent, Yaba, Lagos, Nigeria; 2 Department of Biological Sciences, Federal University Oye, Oye, Ekiti State, Nigeria; 3 World Health Organization, Lagos, Nigeria; 4 National Malaria Elimination Program, Federal Ministry of Health, Abuja, Nigeria; 5 Department of Zoology, University of Ilorin, Ilorin, Kwara State, Nigeria; 6 Community Vision Initiative, Abuja, Nigeria; Institute of Zoology Chinese Academy of Sciences, CHINA

## Abstract

*Anopheles gambiae*, *An*. *coluzzii* and *An*. *arabiensis* are the three major vectors of malaria in Nigeria. These mosquitoes have developed resistance to different insecticides. Insecticides resistance intensity assay was recently introduced to provide insight into the potential operational significance of insecticide resistance. Here, we present data on pyrethroids resistance intensity and resistance mechanisms from six vector surveillance sites (Lagos, Ogun, Edo, Anambra, Kwara and Niger) in Nigeria. Adult *Anopheles* reared from larval collections were tested using WHO insecticides susceptibility protocol with 1x concentration of permethrin and deltamethrin followed with intensity assays with 5x and 10x concentrations of both insecticides. Synergistic and biochemical assays were carried out and underlying resistance mechanisms determined following standard protocols. *Anopheles gambiae* constituted >50% samples tested in five sites. Permethrin and deltamethrin resistance was observed at all the sites. The Kdt50 varied from 15 minutes (CI = 13.6–17.2) in deltamethrin to 42.1 minutes (CI = 39.4–44.1) in permethrin. For both insecticides, Kdt95 was >30 minutes with 25% to 87% post exposure mortality at the different sites. The West Africa knock down resistance (*kdr-w*) mechanism was found at each site. Resistant *An*. *gambiae* from Lagos, Ogun and Niger synergized prior to permethrin or deltamethrin exposure showed significant mortality (89–100%) compared to unsynergized mosquitoes (Lagos, p = 0.031; Ogun, p = 0.025; Niger, p = 0.018). Biochemical analyses revealed significant increased levels of P450 enzymes in resistant *Anopheles gambiae* from Lagos (p = 0.038); Ogun (p = 0.042) and Niger (p = 0.028) in addition to GST in Lagos (p = 0.028) and Ogun (p = 0.033). Overall, the results revealed high pyrethroid resistance associated with increased activities of metabolic enzymes (P450 + GST) in *An*. *gambiae* and *An*. *coluzzii* from Lagos and Ogun. The presence of *kdr* + P450 conferred moderate resistance whereas low resistance was the case where *kdr* was the sole resistance mechanism. Findings thus suggests that elevated levels of cytochrome P450 enzymes together with GST were responsible for high or severe pyrethroid resistance.

## Introduction

The use of long-lasting insecticidal nets (LLINs) is one of the most effective measures for malaria control [1–2]. The basis of this intervention lies in the continuing susceptibility of *Anopheles* mosquito vectors to limited numbers of insecticides. Although new classes and combinations of insecticides are being pilot-tested [3], pyrethroid remains the only class currently in use for LLINs production. Over the past five decades, several accounts of reduced *Anopheles* susceptibility to insecticides, resistance to organophosphates, organochlorine and carbamates have been reported [4–5]. Resistance of *Anopheles* to pyrethroid insecticides is a much more recent development, first reported in *Anopheles gambiae* from Côte d'Ivoire [6] and now wide- spread in West, Central and East Africa [7–8]. Resistance is primarily due to target-site insensitivity arising from a single point mutation often referred to as knock down resistance (kdr) [9–11]. Metabolic resistance mechanisms which are principally associated with three enzyme families (cytochrome p450 mono-oxygenaeses (p450s), carboxylesterases (CEOEs) and glutathione-S-transferases (GSTs), have also been implicated in pyrethroid resistance in many sites in Africa [4,12].

Even though pyrethroid resistance is widespread, its impact on malaria control remains unclear. For instance, a just concluded review [13] was unable to determine if LLINs remain effective in the presence of resistance. To address the challenge of insecticides resistance, the World Health Organization (WHO) updated and expanded insecticide resistance test procedure to include resistance intensity assays [14]. It also initiated the Global Plan for Insecticide Resistance Management (GPIRM) [15] which outlined several strategies on how best to respond to resistance [16]. A key pillar in this effort among others, is timely surveillance for effective vector control and resistance management.

Pyrethroid resistance affecting both *Anopheles gambiae* and *Anopheles coluzzii* is widespread in Nigeria [17–20]. At least 18 out of the 36 States have reported pyrethroid resistance in *Anopheles* vectors [21]. The actual number and spread could be higher because, until recently, there was no in-country coordinated platform for insecticide resistance monitoring to present accurate data. Though indiscriminative use of agricultural insecticides has often been cited as a major factor responsible for the development of resistance [22], the rapid increase in the quantity of insecticides used for malaria control following the scale up of vector control activities have exerted significant increase in insecticide selection pressure in *Anopheles* populations evidenced by increased phenotypic resistance and kdr frequency [21]. The realization that in vector control, surveillance and information on resistance intensity could provide predictive value for decision making, the Nigerian Malaria Elimination Program recently established vector surveillance sites in the six geo-political zones. Here, we present data on levels of pyrethroid resistance with corresponding resistance mechanisms in populations of *Anopheles gambiae* from six vector surveillance sites in Nigeria.

## Methods

### Study areas

**Fig 1:**
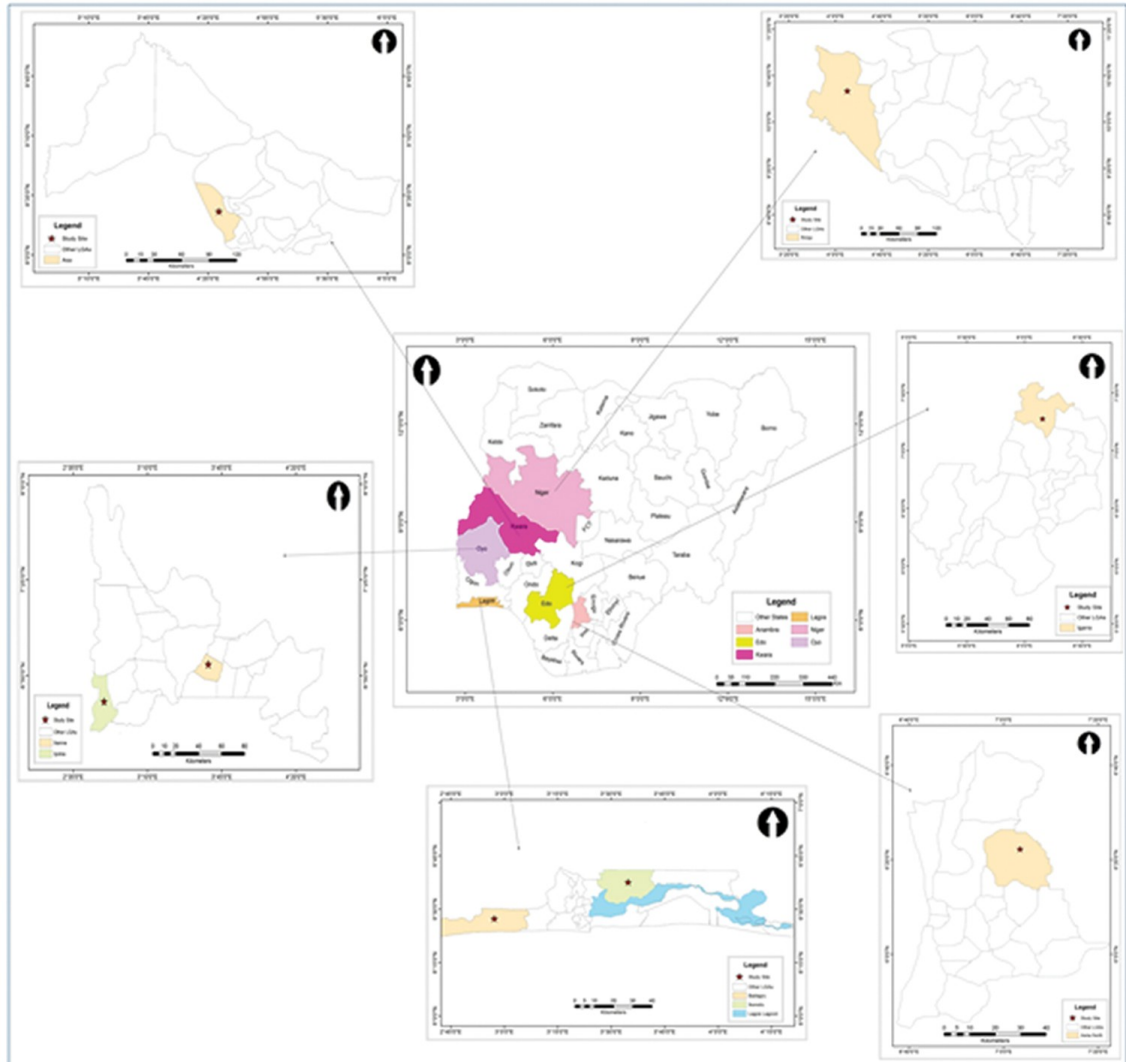
Map of Nigeria showing the study sites in Lagos, Ogun, Edo, Anambra, Kwara and Niger States.

The study was carried out from May to July 2016 in six vector surveillance sites (Fig 1). The use of long lasting insecticidal nets is the main malaria vector control intervention in all the sites in addition to indoor residual spraying in Lagos.

### Mosquitoes collections

*Anopheles* larvae were collected from natural breeding sites by trained Entomology Technicians using standard method [23]. Larvae were sampled on four separate occasions over a period of three weeks. Mosquitoes were reared to adulthood under standard insectary condition (26–27°C and 76–82% relative humidity) at the Nigerian Institute of Medical Research and identified using morphological keys [24–25] and PCR assays [26–27]^,^

### Insecticide susceptibility tests with the standard diagnostic (1x) concentration

Insecticides susceptibility test was carried out using the standard WHO protocol [28] and insecticides test kits supplied by the Vector Control Research Unit, Universiti Sains Malaysia. Two-to three-day old non-blood-fed adult female *Anopheles* were tested. Five replicates of 20–25 mosquitoes were exposed to test papers impregnated with deltamethrin (0.05%) and permethrin (0.75%). Controls included batches of mosquitoes from each site exposed to untreated papers. The knock- down effect of each insecticide was recorded every 10 minutes over the one-hour exposure period. Mosquitoes were then transferred to a recovery tube and provided with 10% glucose solution. Final mortality was recorded 24 hours post-exposure. All batches of insecticide paper used were pre-tested on the Kisumu susceptible laboratory strain of *An*. *gambiae* known to be fully (100%) susceptible to pyrethroid. The tests were carried out at 26–27°C and 76–82% relative humidity.

### Intensity assay

Based on the results of the susceptibility tests with 1x standard diagnostic concentration, intensity tests with x5 concentration of permethrin and deltamethrin were carried out at all the sites. The 10x concentrations of both insecticides was used where applicable according to the updated WHO protocols [14]. The 5x and 10x diagnostic concentrations were prepared using Technical-grade of permethrin and deltamethrin (Sigma-Aldrich) diluted in acetone and olive oil (1:1) as contained in the manufactural instruction to form a stock solution. For each insecticide concentration, 2ml of the stock was applied on filter papers (12 × 15 cm Whatman® no. 1: Whatman International Ltd., England). Filter papers treated with silicone was used as control. All filter papers were dried at room temperature for 24 hours prior to use.

Five replicates of 20–25 mosquitoes were tested for each insecticide concentration (where applicable). The exposures were carried out for the standard one hour period and final mortality recorded after 24-hours holding period during which 10% glucose solution was provided. All *Anopheles gambiae s*.*l*. tested (dead and live mosquitoes) were identified to species level using PCR assays [26–27]. Briefly, DNA was extracted from each mosquito as previously described [26]. PCR amplification was carried out using thermal cycler (Eppendorf, AG Hamburg, Germany) with an initial denaturation at 94 ^o^C for 2 min, followed by 30 cycles of denaturation at 94 ^o^C or 30 s annealing at 50 ^o^C for 30 s and extension at 72 ^o^C for 30 s with a final extension at 72 ^o^C for 8 min. The amplified DNA was separated on 2.5% agarose gel, stained with 1% ethidium bromide and visualized on an ultraviolet violet trans-illuminator.

### Determination of pyrethroid resistance mechanisms

The population of *An*. *gambiae* that survived the 1x standard diagnostic concentration of permethrin at each site was divided into two: the first subset was analyzed together with dead mosquitoes (n = 2038) to species level using PCR [26–27] and for the presence of the *kdr* mutation using allele-specific PCR diagnostic tests designed for the West and East African *kdr* mutations [9,11]. The second subset (n = 330: 50–60 families per site) was induced to lay eggs in the insectary and F1 progeny raised under standard insectary condition (26–27°C and 76–82% relative humidity) used for synergist and biochemical analyses.

Synergist assay with 4% Piperonyl Butoxide (PBO) was performed along with permethrin [29–30]. The F1 progenies that survived 1x concentration of permethrin were further sub-divided into two sub sets. A subset was exposed to 4% PBO prior to permethrin exposure. The second subset was exposed to permethrin only. To determine the role of metabolic degradation as a mechanism for pyrethroid resistance, mortality rate was compared between PBO exposed and unexposed mosquitoes. To investigate the relative role of specific metabolic pathways inhibited by this synergist, enzyme assays were carried out on live mosquitoes not exposed to insecticides were used to measure esterase, glutathione S-transferase (GST) and cytochrome P450 monooxygenase activities as previously described [30]. The same mosquitoes tested for P450 monooxygenase and GST were also tested for esterases activities. All mosquitoes tested were identified to species level by PCR [26–27].

### Data analysis

The knock-down times for 50 and 95% (Kdt50 and Kdt95) mosquito exposed was analyzed using probit-log [31] with MedCalc statistical software version 17.1. Following insecticides tests and PCR identification, data were segregated and analyzed separately for each species using the updated WHO guideline [14]:

- 98–100% mortality at 1x dose confirms resistance
- 98–100% mortality at 5× dose indicates a low resistance intensity. Not necessary to assay at the 10× dose.
- < 98% mortality at 5× dose indicates a moderate resistance intensity. Further test at 10× dose needed.
- 98–100% mortality at 10× dose confirms a moderate resistance intensity.
- < 98% mortality at 10× dose indicates high resistance intensity.

Enzymatic activity of P450 oxidases, GST and esterases were analyzed and compared with the Kisumu reference *Anopheles* strain using ANOVA (F-statistic) with significant p-values < 0.05.

### Ethics

The study was approved by the Research Ethics Review Committee and Institutional Review Board of the Nigerian Institute of Medical Research with protocol approval ref. No. IRB/14/257.

## Results

### Proportion of *Anopheles gambiae*, *An. coluzzii* and *An. arabiensis* in the study sites

The species composition of *Anopheles gambiae s*.*l*. found in this study sites did not differ from previous records with *An*. *gambiae* constituting >50% at each site except in Edo where *An*. *coluzzii* represented 65% (Table 1). Wherever present, *An*. *arabiensis* was < 20% of the *Anopheles* tested. Overall, there were mixed populations of *An*. *gambiae* and *An*. *coluzzii* in Lagos, Ogun, Edo, Kwara and Niger but a pure collection of *An*. *gambiae* in Anambra. No hybrid form of these species was found.

**Table 1:** Number of *Anopheles gambiae* s.l. tested with proportion of *An*. *gambiae*, *An*. *coluzzii* and *An*. *arabiensis*.

State: (site)	N	Number and proportion (%)		
		*An*. *gambiae ss*.	*An*. *coluzzii*	*An*. *arabiensis*
Lagos (Ikorodu-Badagry)	680	405 (59.6)	275 (40.4)	-
Ogun (Ipokia-Irolu)	600	332 (55.3)	207 (34.5)	61 (10.2)
Edo (Igara)	420	147 (35.0)	273 (65.0)	-
Anambra (Achalla)	540	530 (98.1)	-	-
Niger (New Bussa)	540	315 (58.3)	125 (23.2)	100 (18.5)
Kwara (Ayede)	480	300 (62.5)	125 (26.0)	55 (11.5)

### Status of pyrethroid resistance at the study sites

The Kisumu strain used as control was fully susceptible to the standard (1x) diagnostic concentration of permethrin (0.75%) and deltamethrin (0.05%) with 50% knock down recorded within 10 minutes’ exposure to both insecticides and 100% mortality 24-hours’ post exposure. In contrast *An*. *gambiae* s.l. from the six sites were resistant to permethrin and deltamethrin. The knock-down times for 50% mosquitoes (Kdt50) varied from 15 minutes (CI = 13.6–17.2) in *Anopheles* exposed to deltamethrin in Edo to 42.1 minutes (CI = 39.4–44.1) in mosquitoes exposed to permethrin in Lagos. For both insecticides, Kdt95 was > 30 minutes at each site. The 24-hour post exposure mortality from the six sites was 25 to 85% in permethrin (Table 2) and 30 to 87% in deltamethrin (Table 3). The lowest mortality (< 17.0%) and thus highest resistant was recorded in mosquitoes exposed to permethrin in Lagos. Mortality in the control was < 5% for each test.

**Table 2:** Mortality of *Anopheles gambiae* s.l. after 24-hr post exposure to 1x, 5x and 10x concentrations of permethrin in WHO bioassays and associated resistance intensity based on WHO classification [14].

State (site)	Permethrin diagnostic concentration (%)				
		1x (0.75)	5x (3.75)	10x (7.5)	
Lagos: Ikorodu-Badagry	No. exposed	120	100	100	High
	Kdt_50_ (CI) minutes	42.1 (39.4–44.1)	24.2 (22.1–27.2)	18.8(17.1–21.5)	
	Kdt_95_ (CI) minutes	> 60	30.5 (27.5 32.1)	23.2 (21.1–25.8)	
	24-h % mortality	16.7	55.0	89.0	
Ogun: Ipokia-Irolu	No. exposed	100	100	100	High
	Kdt_50_ (CI) minutes	38.9 (37.2–41.2)	22.6 (21.2–26.5)	16.7(15.8–18.6)	
	Kdt_95_ (CI) minutes	49.6 (47.2–53.4)	29.5 (28.5 32.2)	23.5 (22.5–25.9)	
	24-h % mortality	32(32.0)	66(66.0)	95(95.0)	
Edo: Igara	No. exposed	100	100	NA*	Low
	Kdt_50_ (CI) minutes	15.9 (12.7–18.2)	13.2 (12.2–15.5)		
	Kdt_95_ (CI) minutes	30.4 (27.2–32.4)	26.5 (25.3–28.6)		
	24-h % mortality	85.0	100		
Anambra: Achalla	No. exposed	100	100	NA*	Low
	Kdt_50_ (CI) minutes	20.9 (18.8–22.2)	18.2 (16.1–20.5)		
	Kdt_95_ (CI) minutes	38.4 (36.2–41.4)	28.5 (28.0–30.6)		
	24-h % mortality	80	99		
Niger: New Bussa	No. exposed	100	120	110	Moderate
	Kdt_50_ (CI) minutes	27.5 (25.2–30.2)	21.6 (20.2–23.5)	15.8(14.1–17.7)	
	Kdt_95_ (CI) minutes	38.5 (37.2–41.6)	28.6 (27.5 32.0)	20.2 (18.1–22.2)	
	24-h % mortality	65	87.5	98.2	
Kwara: Ayede	No. exposed	120	120	NA*	Low
	Kdt_50_ (CI) minutes	18.8 (16.5–21.2)	16.8 (15.0–19.1)		
	Kdt_95_ (CI) minutes	36.5 (35.2–38.1)	27.5 (26.2–29.4)		
	24-h % mortality	89(74.2)	118(98.3)		

*NA (Not applicable): 98–100% mortality at 5× dose indicates a low resistance intensity. Not necessary to assay at 10x dose.

**Table 3:** Mortality of *Anopheles gambiae* s.l. after 24-hr post exposure to 1x, 5x and 10x concentrations of deltamethrin in WHO bioassays and associated resistance intensity based on WHO classification [14].

Sites	Deltamethrin diagnostic concentration (%)				
		1x (0.05)	5x (0.25)	10x (0.5)	
Lagos: Ikorodu Badagry	No. exposed	120	120	120	High
	Kdt_50_ (CI) minutes	25.8 (23.7–27.2)	18.5 (16.1–21.2)	13.2(12.1–15.8)	
	Kdt_95_ (CI) minutes	38.5 (37.1–40.1)	29.2 (27.4 31.5)	22.1 (19.0–24.6)	
	24-h % mortality	30.0	70.8	93.3	
Ogun: Ipokia-Irolu	No. exposed	100	100	100	Moderate
	Kdt_50_ (CI) minutes	30.8 (27.2–33.2)	20.5 (18.1–23.5)	14.5(12.0–16.2)	
	Kdt_95_ (CI) minutes	42.2 (40.1–44.4)	27.4 (25.2 29.0)	21.1 (19.9–23.7)	
	24-h % mortality	41.0	75.0	99.0	
Edo: Igara	No. exposed	120	100	NA*	Low
	Kdt_50_ (CI) minutes	15.0 (13.6–17.2)	13.0 (12.8–15.1)		
	Kdt_95_ (CI) minutes	30.8 (27.0–32.2)	25.8 (24.3–27.4)		
	24-h % mortality	87.5	100		
Anambra: Achalla	No. exposed	120	120	NA*	Low
	Kdt_50_ (CI) minutes	18.5 (16.8–21.2)	16.2 (14.1–17.9)		
	Kdt_95_ (CI) minutes	32.4 (30.2–33.1)	25.2 (24.3–27.6)		
	24-h % mortality	76.7	98.3		
Niger: New Bussa	No. exposed	120	120	110	High
	Kdt_50_ (CI) minutes	25.3 (24.2–27.2)	19.6 (17.4–22.5)	13.8(11.8–15.4)	
	Kdt_95_ (CI) minutes	39.2 (37.0–42.8)	27.6 (25.5 28.8)	19.5 (18.0–21.2)	
	24-h % mortality	61.7	85.0	94.5	
Kwara: Ayede	No. exposed	120	120	NA*	Low
	Kdt_50_ (CI) minutes	17.8 (15.2–19.8)	14.2 (12.5–16.1)		
	Kdt_95_ (CI) minutes	30.4 (28.2–32.1)	26.4 (24.9–29.1)		
	24-h % mortality	76.7	100		

*NA (Not applicable): 98–100% mortality at 5× dose indicates a low resistance intensity. Not necessary to assay at 10x dose.

### Resistance intensity

**Fig 2:**
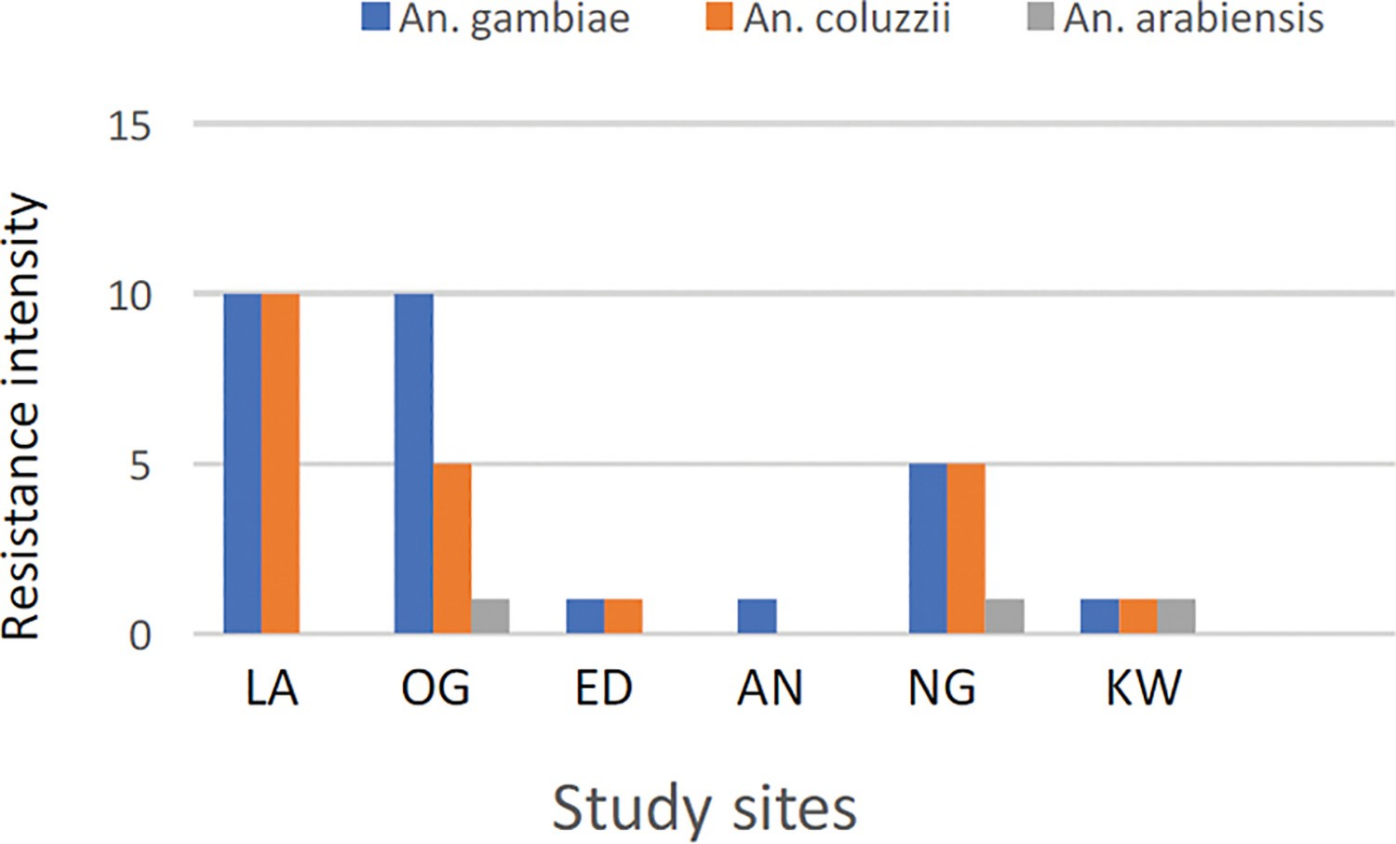
Permethrin resistance intensity in *Anopheles gambiae*, *An*. *coluzzii* and *An*. *arabiensis* from Lagos (LA), Ogun (OG), Edo (ED), Anambra (AN), Niger (NG) and Kwara (KW).

**Fig 3:**
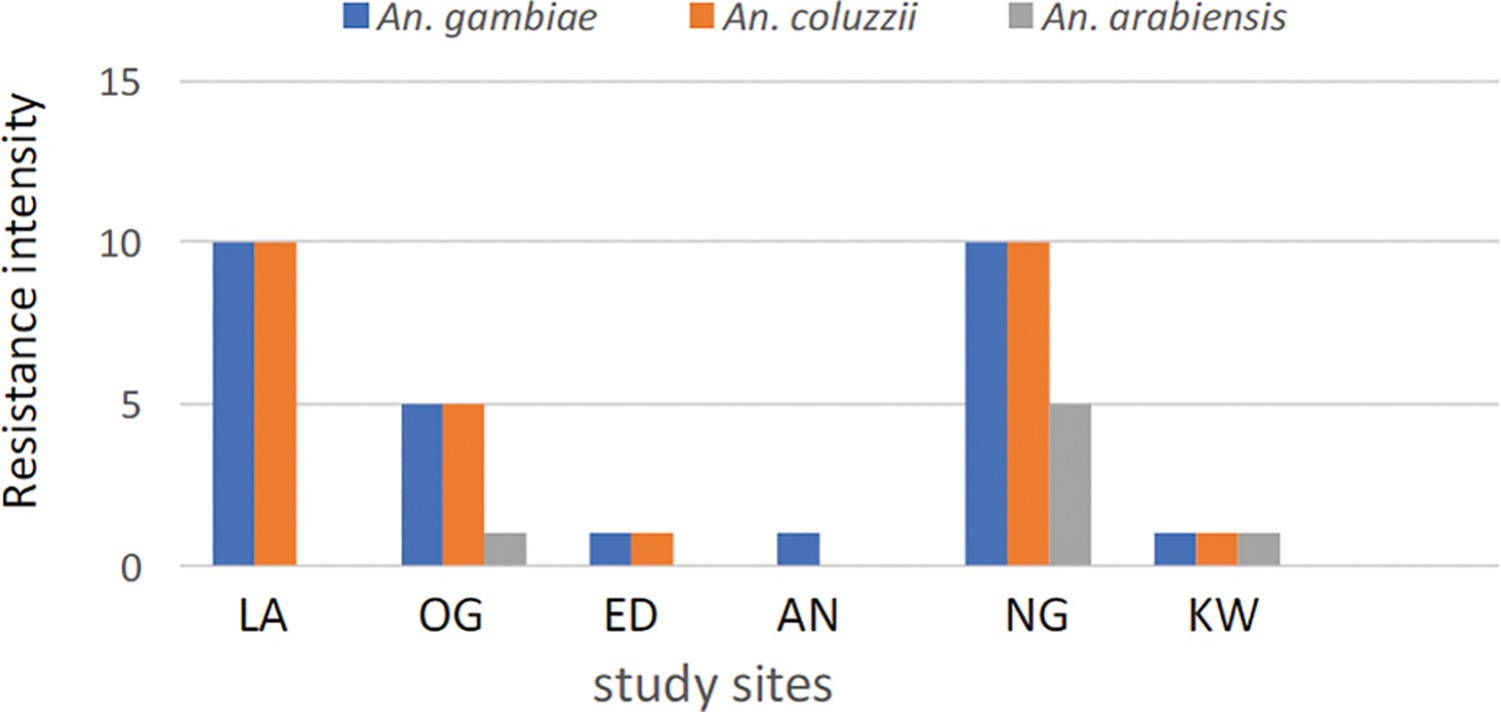
Deltamethrin resistance intensity in *Anopheles gambiae*, *An*. *coluzzii* and *An*. *arabiensis* from Lagos (LA), Ogun (OG), Edo (ED), Anambra (AN), Niger (NG) and Kwara (KW).

The results of the intensity assays with permethrin and deltamethrin are presented in Tables 2 and 3. The Kdt50 and Kdt95 values for the two insecticides indicate differential tolerance levels of *An*. *gambiae s*.*l*. to pyrethroid in the study sites. More than two-fold increase (from 1x to 10x) in the tolerance level to permethrin and deltamethrin was observed at three sites (Lagos, Ogun and Niger). Based on the expanded WHO classification, there was high permethrin and deltamethrin resistance in Lagos, moderate deltamethrin and high permethrin resistance in Ogun but moderate permethrin and high deltamethrin resistance in Niger. In contrast, resistance was low for both insecticides in Edo, Anambra and Kwara (Tables 2 and 3). Species specific analysis revealed high permethrin and deltamethrin resistance in *An*. *gambiae* and *An*. *coluzzii* from Lagos (Fig 2) in addition to deltamethrin in Niger (Fig 3). Where present, percentage mortality data of *An*. *arabiensis* against 5x concentration of both pyrethroid insecticides showed low resistance (S1 and S2 Tables).

### Pyrethroid resistance mechanisms

The West Africa *Kdr* mutation (*Kdr-w*) was confirmed in *An*. *gambiae* at all the study sites with frequencies ranging from 55% in Anambra to 88% in Niger (S3 Table). The overall *kdr-w* frequency was significantly higher only in Niger (χ^2^ = 0.379, df = 5, P = 0.024). The East African mutation (*Kdr-e*) was absent in the mosquitoes tested.

**Fig 4:**
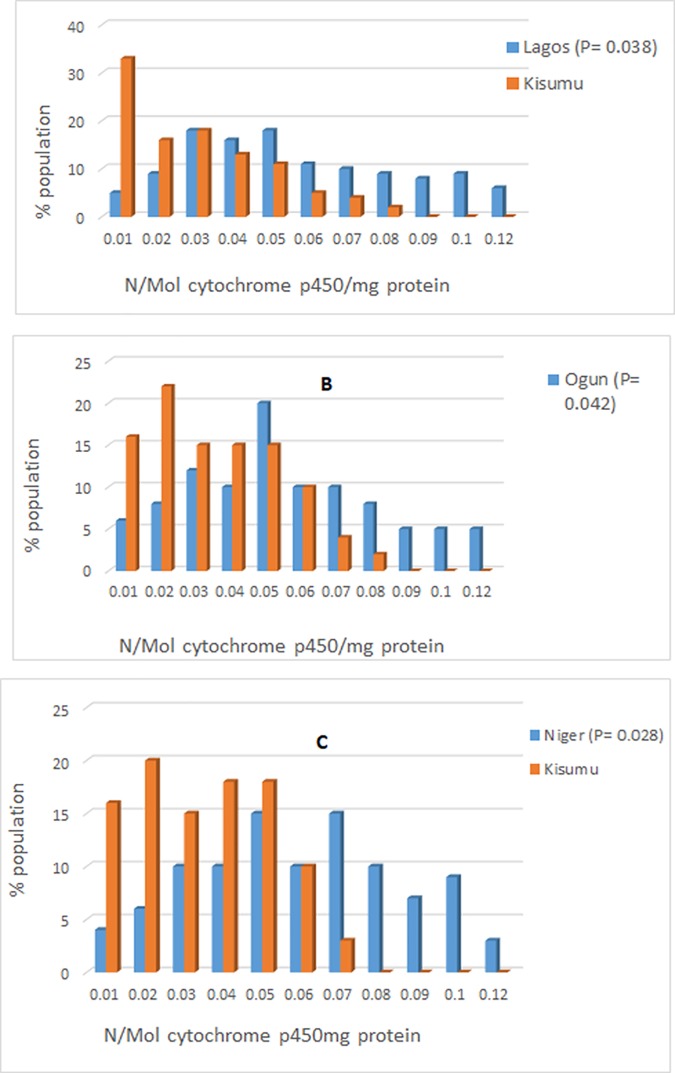
Mean level of monooxygenase in pyrethroid resistant *Anopheles gambiae* from Lagos (A), Ogun (B) and Niger (C) in relation to the susceptible Kisumu strain.

**Fig 5:**
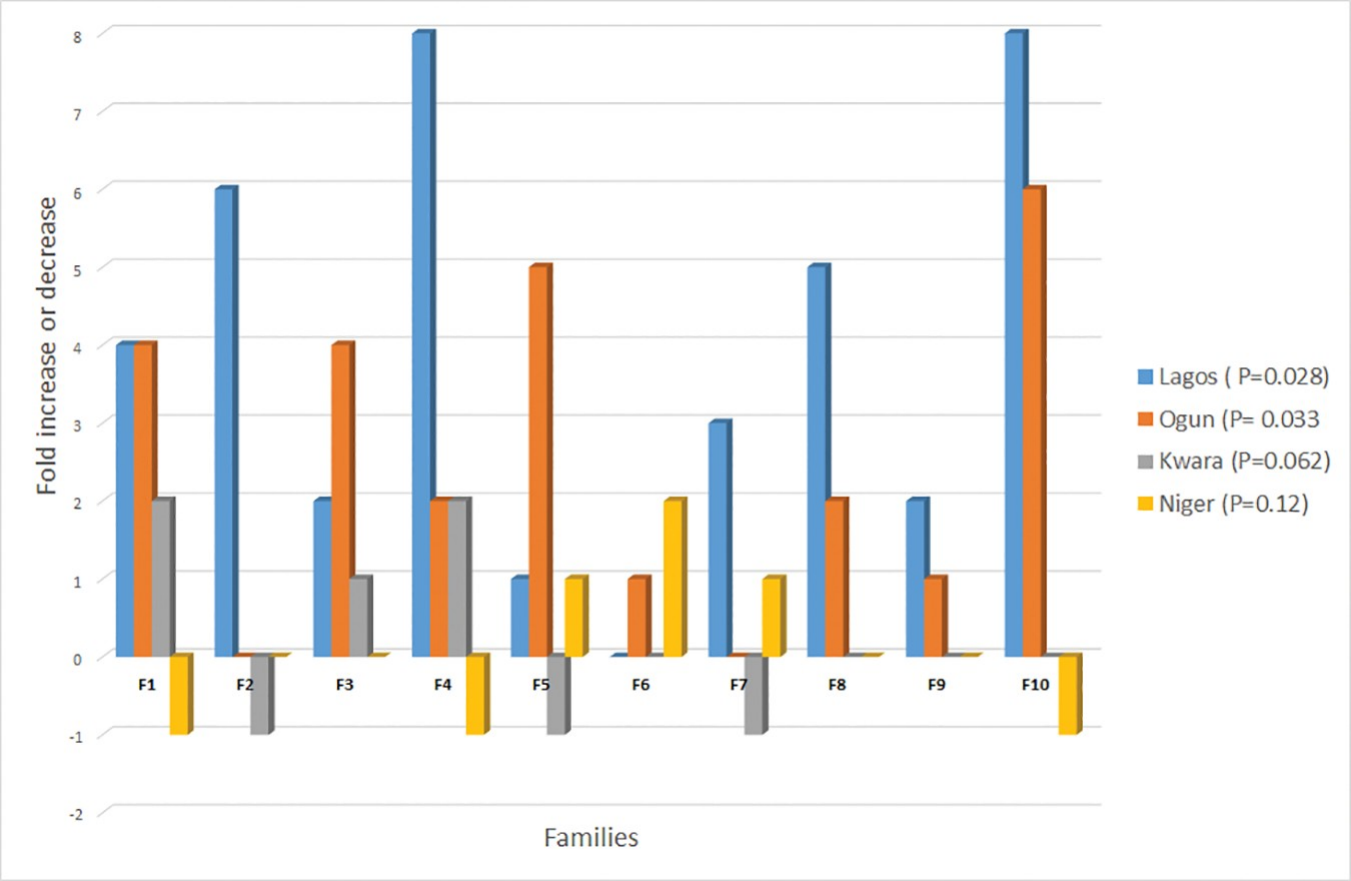
Percentage increase or decrease in GST activity level in families of *Anopheles gambiae* from Lagos, Ogun, Kwara and Niger relative to the baseline Kisumu level set at zero.

Resistant *An*. *gambiae* populations from Lagos, Ogun and Niger synergized prior to permethrin or deltamethrin exposure showed significant mortality (89–100%) compared to unsynergized mosquitoes (Lagos, p = 0.031; Ogun, p = 0.025; Niger p = 0.018) (S4 and S5 Tables). Biochemical analysis revealed increased levels of P450 activity (Fig 4) with mean level of monooxygase > 0.06 N/mol p450 protein compared with the standard Kisumu strain (Lagos, F = 3.48, df = 39, p = 0.038; Ogun, F = 4.25, df = 39, p = 0.042; Niger, F = 5.40, df = 39, p = 0.028). The mean P450 activity in resistant *An*. *gambiae* from the remaining sites were like that of the Kisumu strain. Furthermore, GST activities (Fig 5) were significantly higher in resistant populations of *An*. *gambiae* from Lagos (p = 0.028) and Ogun (P = 0.033) compared to that of the reference Kisumu strain. In contrast, the mean esterase activity in mosquitoes from all the sites were like the Kisumu reference strain indicating the absence of esterase as a resistance mechanism in the mosquito populations tested.

## Discussion

The study set out to determine pyrethroid resistance intensity in areas where *Anopheles gambiae* is resistant to insecticides. The species composition of *An*. *gambiae* s.l. found in this study did not differ from previous records [17–19] with a predominance of *An*. *gambiae* and *An*. *coluzzii*. The high pyrethroid resistance intensity in *An*. *gambiae* from Lagos, Ogun and Niger is not surprising considering the prevailing insecticide selection pressure on vector populations following the rapid scale up and use of pyrethroid-based vector control interventions and agricultural usage of pyrethroid insecticides in these areas. Similar resistance intensity (10x) was recently reported in different laboratory strains of *An*. *gambiae* from Southern Africa [32].

The present results clearly show the differential effect of the same class of insecticide on three *Anopheles* species: *An*. *gambiae*, *An*. *coluzzii* and *An*. *arabiensis*. In previous studies [17–18], we investigated pyrethroid susceptibility levels of *An*. *gambiae* and *An*. *arabiensis* from Lagos and Niger and found that *An*. *arabiensis* was fully susceptible to permethrin and deltamethrin. This contrast the present observations in Kwara and Niger where *An*. *arabiensis* is resistant to deltamethrin. Pyrethroid resistance in *An*. *arabiensis* is not common in Nigeria [19] and there is currently no published information on its spread nor underlying resistance mechanisms. Despite the low resistance, pyrethroid resistance in *An*. *arabiensis* could be much wider spread than previously thought.

Most insecticide-resistance monitoring projects emphasize resistance detection, identification of underlying resistance mechanisms and spread. The *Kdr*-mediated resistance is one of the most common mechanisms reported in pyrethroid resistance *Anopheles* populations. The role of metabolic detoxification of pyrethroids is increasingly being investigated in *Anopheles* across different sites in Africa. The study revealed a significant increase in the levels of P450 and GST activities in *An*. *gambiae* and *An*. *coluzzii* from Lagos and Ogun which consolidated findings from previous study [33] on multiple pyrethroid resistance mechanisms in *An*. *gambiae* from Nigeria. Thus, the operational significance of resistance could hinge on interplay between different resistance mechanisms in the vector population. Interestingly, in Edo and Anambra where *kdr* is present as the sole resistance mechanism, it appears to be associated with low resistance in the *Anopheles* populations. In all, three scenarios emerged from the study. In Lagos and Ogun with high pyrethroid resistance intensity, *kdr* plus metabolic: P450s and GST are the main mechanisms; moderate to high pyrethroid resistance intensity was observed in Niger in the presence of P450 plus *Kdr*; lastly, *kdr* was the only resistance mechanism in Edo and Anambra where permethrin and deltamethrin resistance was low. Although the operational significance of the severe pyrethroid resistance recorded in the study is uncertain, similar pyrethroid resistance phenotype in southern African *An*. *funestus* has been shown to have serious operational implications for malaria vector control in that region [34–36]. Therefore, a much more detailed entomological and epidemiological investigations will be required to unravel the impact of high resistance intensity on malaria control in Nigeria.

Summarily, the study revealed a probable association between resistance intensity and multiple resistance mechanisms and suggest that elevated levels of P450 together with GST were responsible for high pyrethroid resistance. Accordingly, resistance intensity could provide useful information on plausible mechanism developed by the local malaria vectors to combat pyrethroid insecticides.

## Supporting information

S1 Table Number of *Anopheles gambiae*, *Anopheles coluzzii* and *Anopheles arabiensis* in test population, proportion knock down and 24-hr post exposure mortality after exposure to 1x, 5x and 10x concentrations of permethrin in WHO bioassays. (DOCX)

S2 Table Number of *Anopheles gambiae*, *Anopheles coluzzii* and *Anopheles arabiensis* in test population, proportion knock down and 24-hr post exposure mortality after exposure to 1x, 5x and 10x concentrations of deltamethrin in WHO bioassays. (DOCX)

S3 Table Genotype count and frequency of the West Africa knock down resistance mutation in *Anopheles gambiae* and *Anopheles coluzzii*. (DOCX)

S4 Table Synergist assay: Knock down and 24 hr mortality of *Anopheles gambiae* exposed to permethrin (0.75%) only compared with permethrin (0.75%) + PBO in WHO bioassays. (DOCX)

S5 Table Synergist assay: Knock down and 24 hr mortality of *Anopheles gambiae* exposed to deltamethrin (0.05%) only compared with deltamethrin (0.05%) + PBO (4%) in WHO bioassays. (DOCX)
