# Supplementary material for: Pyrethroids resistance intensity and resistance mechanisms in Anopheles gambiae from malaria vector surveillance sites in Nigeria
Source: PLoS One. 2018 Dec 5;13(12):e0205230. doi: 10.1371/journal.pone.0205230 (PMC6281219; doi:10.1371/journal.pone.0205230)
Supplement: S1 Table — (DOCX) [file pone.0205230.s001.docx]

S1 Table. Number of *Anopheles gambiae*, *Anopheles coluzzii* and *Anopheles arabiensis* in test population, proportion knock down and 24-hr post exposure mortality after exposure to 1x, 5x and 10x concentrations of permethrin in WHO bioassays

| Sites | Total no. exposed | *Anopheles* species in test population exposed to different permethrin concentration | | | | | | | | |
| --- | --- | --- | --- | --- | --- | --- | --- | --- | --- | --- |
|  |  | 1x concentration (0.75%) | | | 5x concentration (3.75%) | | | 10x concentration (7.5%) | | |
|  |  | *gambiae* | *coluzzii* | *arabbiensis* | *gambiae* | *Coluzzii* | *arabbiensis* | *gambiae* | *coluzzii* | *arabbiensis* |
| Lagos | n=320 | 80 | 40 | - | 65 | 35 | - | 62 | 38 | - |
|  | No. (%) k- down | 40(50.0) | 25 (62.5) | - | 40 (61.5) | 26 (74.3) | - | 41 (66.1) | 29 (76.3) | - |
|  | 24-h % mortality | 12 (15.0) | 8 (20.0) | - | 32 (49.2) | 22 (62.9) | - | 53 (85.5) | 36  (94.7) | - |
| Ogun | n=300 | 60 | 30 | 10 | 62 | 34 | 4 | 65 | 35 | 0 |
|  | No. (%) k- down | 7 (11.7) | 15 (50.0) | 10 (100.0) | 38 (61.2) | 29 (85.3) | 4 (100.0) | 59 (90.8) | 35 (100.0) | 0 |
|  | 24-h % mortality | 6 (10.0) | 16 (53.3) | 10 (100.0) | 32  (51.6) | 30  (88.2) | 4  (100.0) | 60  (92.3) | 35 (100.0) | 0 |
| Edo | n=200 | 38 | 62 | - | 39 | 61 | - | - | - | - |
|  | No. (%) k- down | 23 (60.5) | 62 (100.0) | - | 39 (100.0) | 61 (100.0) | - | - | - | - |
|  | 24-h % mortality | 23 (60.5) | 62 (100.0) | - | 39 (100.0) | 61 (100.0) | - | - | - | - |
| Anambra | n=200 | 100 | - | - | 100 | - | - | - | - | - |
|  | No. (%) k- down | 74  (74.0) | - | - | 86 (86.0) | - | - | - | - | - |
|  | 24-h % mortality | 80 (80.0) | - | - | 99 (99.0) | - | - | - | - | - |
| Niger | n=330 | 60 | 25 | 15 | 72 | 27 | 21 | 65 | 45 | 0 |
|  | No. (%) k- down | 30 (50.0) | 17 (68.0) | 15 (100.0) | 45 (62.5) | 23 (85.2) | 21 (100.0) | 63  (96.9) | 45  (100.0) | 0 |
|  | 24-h % mortality | 32  (53.3) | 18  (72.0) | 15 (100.0) | 59 (81.9) | 25 (92.6) | 21 (100.0) | 65  (100.0) | 45  (100.0) | 0 |
| Kwara | n=240 | 80 | 25 | 15 | 82 | 27 | 11 | - | - | - |
|  | No. (%) k- down | 52 (65.0) | 21 (84.0) | 15 (100.0) | 75  (91.5) | 27 (100.0) | 11 (100.0) | - | - | - |
