# Supplementary material for: Pyrethroids resistance intensity and resistance mechanisms in Anopheles gambiae from malaria vector surveillance sites in Nigeria
Source: PLoS One. 2018 Dec 5;13(12):e0205230. doi: 10.1371/journal.pone.0205230 (PMC6281219; doi:10.1371/journal.pone.0205230)
Supplement: S2 Table — (DOCX) [file pone.0205230.s002.docx]

S2 Table. Number of *Anopheles gambiae*, *Anopheles coluzzii* and *Anopheles arabiensis* in test population, proportion knock down and 24-hr post exposure mortality after exposure to 1x, 5x and 10x concentrations of deltamethrin in WHO bioassays

| Sites | Total no. exposed | *Anopheles* species in test population exposed to different deltamethrin concentrations | | | | | | | | |
| --- | --- | --- | --- | --- | --- | --- | --- | --- | --- | --- |
|  |  | 1x concentration (0.05%) | | | 5x concentration (0.25%) | | | 10x concentration (0.5%) | | |
|  |  | *gambiae* | *coluzzii* | *Arabbiensis* | *gambiae* | *Coluzzii* | *arabbiensis* | *gambiae* | *coluzzii* | *arabbiensis* |
| Lagos | n=360 | 80 | 40 |  | 85 | 35 | - | 79 | 41 | - |
|  | No. (%) k- down | 38(47.5) | 28 (70.0) | - | 45 (52.9) | 28(80.0) | - | 69 (87.3) | 37 (90.2) | - |
|  | 24-h % mortality | 16 (20.0) | 20 (50.0) | - | 50 (58.8) | 35(100.0) | - | 73 (92.4) | 39  (95.1) | - |
| Ogun | n=320 | 62 | 38 | 0 | 60 | 34 | 6 | 73 | 47 | 0 |
|  | No. (%) k- down | 15 (24.2) | 25 (65.8) | 0 | 38 (63.3) | 29 (85.3) | 6 (100.0) | 59 (80.8) | 47 (100.0) | 0 |
|  | 24-h % mortality | 16 (25.8) | 25 (65.8) | 0 | 45  (75.0) | 30  (88.2) | 6 (100.0) | 73  (100.0) | 47  (100.0) | 0 |
| Edo | n=220 | 55 | 65 | - | 35 | 65 | - | - | - | - |
|  | No. (%) k- down | 28 (50.9) | 62 (95.4) | - | 30 (85.7) | 64 (98.5) | - | - | - | - |
|  | 24-h % mortality | 47 (85.5) | 58 (93.5) | - | 35 (100.0) | 65 (100.0) | - | - | - | - |
| Anambra | n=240 | 120 | - | - | 120 | - | - | - | - | - |
|  | No. (%) k- down | 74  (61.7) | - | - | 96 (80.0) | - | - | - | - | - |
|  | 24-h % mortality | 92 (76.7) | - | - | 119 (99.2) | - | - | - | - | - |
| Niger | n=350 | 68 | 32 | 20 | 75 | 25 | 20 | 70 | 40 | 0 |
|  | No. (%) k- down | 30 (44.1) | 17 (53.1) | 15  (75.0) | 45 (60.0) | 20  (80.0) | 15  (75.0) | 67 (95.7) | 37 (92.5) | 0 |
|  | 24-h % mortality | 26  (38.2) | 28  (87.5) | 17  (85.0) | 57  (76.0) | 20  (80.0) | 18  (90.0) | 67 (95.7) | 37 (92.5) | 0 |
| Kwara | n=240 | 68 | 30 | 22 | 75 | 25 | 20 | - | - | - |
|  | No. (%) k- down | 54 (79.4) | 25 (83.3) | 15 (68.1) | 70  (93.3) | 25 (100.0) | 20 (100.0) | - | - | - |
|  | 24-h % mortality | 53 (77.9) | 24 (80.0) | 15 (68.1) | 74 (98.7) | 25 (100.0) | 20  (100.0) | - | - | - |
