# Supplementary material for: Pyrethroids resistance intensity and resistance mechanisms in Anopheles gambiae from malaria vector surveillance sites in Nigeria
Source: PLoS One. 2018 Dec 5;13(12):e0205230. doi: 10.1371/journal.pone.0205230 (PMC6281219; doi:10.1371/journal.pone.0205230)
Supplement: S3 Table — (DOCX) [file pone.0205230.s003.docx]

S3 Table. Genotype count and frequency of the West Africa knock down resistance mutation in *Anopheles gambiae* and *Anopheles coluzzii*

| Sites | Genotype count and frequency (%) of the kdr-w mutation (R) | | | | | | | | | |
| --- | --- | --- | --- | --- | --- | --- | --- | --- | --- | --- |
|  | *Anopheles gambiae* | | | | | *Anopheles coluzzii* | | | | |
|  | **n** | **RR** | **Rs** | **Ss** | **% Kdr frequency** | **N** | **RR** | **Rs** | **Ss** | **% Kdr frequency** |
| Lagos | **405** | 102 | 133 | 170 | 58.0 | **275** | 25 | 39 | 211 | 23.3 |
| Ogun | **341** | 100 | 88 | 153 | 55.1 | **207** | 15 | 11 | 181 | 12.6 |
| Edo | **147** | 30 | 53 | 64 | 56.5 | **273** | 12 | 17 | 244 | 10.6 |
| Anambra | **530** | 100 | 191 | 239 | 54.9 | **-** | - | - | - | - |
| Niger | **315** | 120 | 158 | 37 | 88.2 | **125** | 12 | 19 | 94 | 24.8 |
| Kwara | **300** | 130 | 95 | 75 | 75.0 | **125** | 7 | 12 | 106 | 15.2 |
