# Supplementary material for: Pyrethroids resistance intensity and resistance mechanisms in Anopheles gambiae from malaria vector surveillance sites in Nigeria
Source: PLoS One. 2018 Dec 5;13(12):e0205230. doi: 10.1371/journal.pone.0205230 (PMC6281219; doi:10.1371/journal.pone.0205230)
Supplement: S4 Table — (DOCX) [file pone.0205230.s004.docx]

S4 Table. Synergist assay: knock down and 24 hr mortality of *Anopheles gambiae* exposed to permethrin (0.75%) only compared with permethrin (0.75%) + PBO in WHO bioassays

| Sites |  | Permethrin (0.75%) only | Permethrin (0.75%) + PBO |
| --- | --- | --- | --- |
| Lagos | No. exposed | 120 | 120 |
|  | No. (%) knock down | 68 (56.7) | 99 (82.5) |
|  | 24-hr % mortality | 20 (16.7) | 107 (89.2) |
| Ogun | No. exposed | 100 | 100 |
|  | No. (%) knock down | 45 (45.0) | 89 (89.0) |
|  | 24-hr % mortality | 32 (32.0) | 95 (95.0) |
| Edo | No. exposed | 100 | 100 |
|  | No. (%) knock down | 78 (78.0) | 76 (76.0) |
|  | 24-hr % mortality | 85 (85.0) | 84 (84.0) |
| Anambra | No. exposed | 100 | 100 |
|  | No. (%) knock down | 79 (79.0) | 76 (76.0) |
|  | 24-hr % mortality | 80 (80.0) | 82 (82.0) |
| Niger | No. exposed | 100 | 100 |
|  | No. (%) knock down | 62 (62.0) | 90 (90.0) |
|  | 24-hr % mortality | 65 (65.0) | 100 (100) |
| Kwara | No. exposed | 120 | 120 |
|  | No. (%) knock down | 79 (65.8) | 82 (68.3) |
|  | 24-hr % mortality | 89 (74.2) | 94 (78.3) |
