# Supplementary material for: Pyrethroids resistance intensity and resistance mechanisms in Anopheles gambiae from malaria vector surveillance sites in Nigeria
Source: PLoS One. 2018 Dec 5;13(12):e0205230. doi: 10.1371/journal.pone.0205230 (PMC6281219; doi:10.1371/journal.pone.0205230)
Supplement: S5 Table — (DOCX) [file pone.0205230.s005.docx]

S5 Table. Synergist assay: knock down and 24 hr mortality of *Anopheles gambiae* exposed to deltamethrin (0.05%) only compared with deltamethrin (0.05%) + PBO (4%) in WHO bioassays

| Sites |  | Deltamethrin 1x (0.05%)only | Deltamethrin 1x (0.05%) + PBO (4%) |
| --- | --- | --- | --- |
| Lagos | No. exposed | 120 | 120 |
|  | No. (%) knock down | 42 (35.0) | 104 (86.7) |
|  | 24-hr % mortality | 30 | 90.0 |
| Ogun | No. exposed | 100 | 100 |
|  | No. (%) knock down | 50 (50.0) | 90 (90.0) |
|  | 24-hr % mortality | 41 | 92.0 |
| Edo | No. exposed | 120 | 100 |
|  | No. (%) knock down | 110 (91.7) | 100 (100) |
|  | 24-hr % mortality | 87.5 | 100.0 |
| Anambra | No. exposed | 120 | 100 |
|  | No. (%) knock down | 96 (80.0) | 88 (88.0) |
|  | 24-hr % mortality | 76.7 | 88.0 |
| Niger | No. exposed | 120 | 100 |
|  | No. (%) knock down | 87 (72.5) | 92 (92.0) |
|  | 24-hr % mortality | 76.7 | 98.0 |
| Kwara | No. exposed | 120 | 100 |
|  | No. (%) knock down | 70 (58.3) | 90 (90.0) |
|  | 24-hr % mortality | 61.7 | 85.0 |
